# Endophytic Lifestyle of Global Clones of Extended-Spectrum β-Lactamase-Producing Priority Pathogens in Fresh Vegetables: a Trojan Horse Strategy Favoring Human Colonization?

**DOI:** 10.1128/mSystems.01125-20

**Published:** 2021-02-09

**Authors:** Ralf Lopes, Danny Fuentes-Castillo, Herrison Fontana, Larissa Rodrigues, Karine Dantas, Louise Cerdeira, Isabel Henriques, Nilton Lincopan

**Affiliations:** a Department of Microbiology, Institute of Biomedical Sciences, University of São Paulo, São Paulo, Brazil; b Department of Pathology, School of Veterinary Medicine and Animal Sciences, University of São Paulo, São Paulo, Brazil; c One Health Brazilian Resistance Project (OneBR), São Paulo, Brazil; d Department of Clinical Analysis, School of Pharmacy, University of São Paulo, São Paulo, Brazil; e Department of Infectious Diseases, Central Clinical School, Monash University, Melbourne, Australia; f Centre for Environmental and Marine Studies (CESAM), University of Aveiro, Aveiro, Portugal; g Department of Life Sciences, Faculty of Sciences and Technology, University of Coimbra, Coimbra, Portugal; University of Illinois at Chicago

**Keywords:** *E. coli* ST648, *E. coli* ST38, *K. pneumoniae* CC307, CTX-M-15, food, One Health, ESBL

## Abstract

The global spread of antibiotic-resistant bacteria and their resistance genes is a critical issue that is no longer restricted to hospital settings, but also represents a growing problem involving environmental and food safety. In this study, we have performed a microbiological and genomic investigation of critical priority pathogens resistant to broad-spectrum cephalosporins and showing endophytic lifestyles in fresh vegetables sold in a country with high endemicity of extended-spectrum β-lactamases (ESBLs). We report the isolation of international high-risk clones of CTX-M-15-producing Escherichia coli, belonging to clonal complexes CC38 and CC648, and Klebsiella pneumoniae of complex CC307 from macerated tissue of surface-sterilized leaves of spinach, cabbage, arugula, and lettuce. Regardless of species, all ESBL-positive isolates were able to endophytically colonize common bean (Phaseolus vulgaris) seedlings, showed resistance to acid pH, and had a multidrug-resistant (MDR) profile to clinically relevant antibiotics (i.e., broad-spectrum cephalosporins, aminoglycosides, and fluoroquinolones). Genomic analysis of CTX-M-producing endophytic *Enterobacterales* revealed a wide resistome (antibiotics, biocides, disinfectants, and pesticides) and virulome, and genes for endophytic fitness and for withstanding acidic conditions. Transferable IncFIB and IncHI2A plasmids carried *bla*_CTX-M-15_ genes and, additionally, an IncFIB plasmid (named pKP301cro) also harbored genes encoding resistance to heavy metals. These data support the hypothesis that fresh vegetables marketed for consumption can act as a figurative Trojan horse for the hidden spread of international clones of critical WHO priority pathogens producing ESBLs, and/or their resistance genes, to humans and other animals, which is a critical issue within a food safety and broader public and environmental health perspective.

**IMPORTANCE** Extended-spectrum β-lactamases (ESBL)-producing *Enterobacterales* are a leading cause of human and animal infections, being classified as critical priority pathogens by the World Health Organization. Epidemiological studies have shown that spread of ESBL-producing bacteria is not a problem restricted to hospitals, but also represents a growing problem involving environmental and food safety. In this regard, CTX-M-type β-lactamases have become the most widely distributed and clinically relevant ESBLs worldwide. Here, we have investigated the occurrence and genomic features of ESBL-producing *Enterobacterales* in surface-sterilized fresh vegetables. We have uncovered that international high-risk clones of CTX-M-15-producing Escherichia coli and Klebsiella pneumoniae harboring a wide resistome and virulome, carry additional genes for endophytic fitness and resistance to acidic conditions. Furthermore, we have demonstrated that these CTX-M-15-positive isolates are able to endophytically colonize plant tissues. Therefore, we believe that fresh vegetables can act as a figurative Trojan horse for the hidden spread of critical priority pathogens exhibiting endophytic lifestyles.

## Abstract (precis)

Extended-spectrum β-lactamases (ESBL)-producing *Enterobacterales* are a leading cause of human and animal infections, being classified as critical priority pathogens by the World Health Organization. Epidemiological studies have shown that spread of ESBL-producing bacteria is not a problem restricted to hospitals, but also represents a growing problem involving environmental and food safety.

## INTRODUCTION

Extended-spectrum β-lactamase (ESBL)-producing Gram-negative bacteria are a leading cause of human and animal infection, being categorized as critical priority pathogens by the World Health Organization (1). In this regard, plasmid-mediated ESBLs of the CTX-M family have been widely identified in different genera of *Enterobacterales*, with CTX-M-15 being the most clinically relevant ESBL worldwide (2). Curiously, *Kluyvera* species, bacteria commonly found in the rhizosphere and endophytic ecosystems, have been proposed as the original source of *bla*_CTX-M_-type genes (3). Therefore, endophytic bacteria that colonize internal tissues of vegetables can represent a hidden mode of transmission of virulent and/or antibiotic-resistant bacteria and their resistance genes to humans and other animals (4–7).

Currently, epidemiological studies have shown that the spread of CTX-M-producing bacteria is not a problem restricted to hospitals, but also represents a growing problem involving environmental and food safety (2). On the other hand, rates of CTX-M-producing *Enterobacterales* in community-acquired urinary tract infections (UTIs) and community fecal carriage have increased significantly worldwide, with developing countries being the most affected (8, 9). In this regard, various factors, such as environmental sources, international travel, and wild, companion, and food-producing animals, have accelerated the global spread of CTX-M ESBLs in the community, mainly in countries with endemic status (8, 10–12).

Specifically, contamination of fresh vegetables by critical priority pathogens is the greatest concern (6, 7, 13, 14), since these foods are consumed raw and this increases the risk of human exposure to ESBL producers and other antibiotic-resistant bacteria with clinical interest (15). Although ingestion of ESBL-producing bacteria may not have an immediate clinical health implication, colonization by this pathway can contribute to the transfer of antibiotic resistance genes to other bacterial species present in the gut microbiota (7, 14, 16). Consequently, a potential threat to human health would be associated with future endogenous infections, mainly in immunosuppressed patients, where therapeutic failure could occur.

Even though clinically significant ESBL-producing *Enterobacterales*, such as Escherichia coli, Klebsiella pneumoniae, and Enterobacter cloacae, have been frequently found as epiphytes on fresh vegetables (13, 14), little is known about their endophytic existence. Therefore, we have performed a microbiological and genomic investigation of critical priority pathogens displaying resistance to broad-spectrum cephalosporins and showing endophytic lifestyles in fresh vegetables sold in a country with high endemicity of ESBLs.

## RESULTS

### Multidrug-resistant ESBL-producing endophytic *Enterobacterales* isolated from fresh vegetables.

The presence of endophytic ESBL-producing *Enterobacterales* was confirmed in 10.4% of 48 fresh vegetables samples screened after surface sterilization, including spinach (2 positive samples for ESBL-producing E. coli strain ESP110 and E. cloacae strain ESP151), cabbage (1 positive sample for 2 ESBL-producing E. coli strains [REP215 and REP237]), lettuce (1 positive sample for ESBL-producing K. pneumoniae [strain ALF301]), and arugula (1 positive sample for ESBL-producing K. pneumoniae [strain RUC232]). All strains displayed a multidrug-resistant profile (17) with high MIC values above resistance breakpoints for broad-spectrum cephalosporins (Table 1). Further resistance to fluoroquinolones was detected in K. pneumoniae RUC232 and E. coli REP215 strains, whereas all endophytic ESBL-positive strains remained susceptible to carbapenems (i.e., imipenem, meropenem, and ertapenem) and amikacin (Table 1).

**TABLE 1:** Antimicrobial resistance profile of endophytic ESBL (CTX-M-15)-producing *Enterobacterales* from commercial vegetables

Strain	Vegetable	Geographical coordinates^*a*^	MIC (μg/ml)^*b*^																			
			Ptz	Cef	Cro	Caz	Fep	Fox	Atm	Ipm	Mem	Etp	Gen	Ami	Nal	Cip	Enr	Lvx	Mxf	Sxt	Chl	Tet
E. cloacae ESP151	Spinach	23°23′24.0″ S; 47°07'48.0″ W	**≥256**	**≥256**	**≥32**	8	12	**128**	**32**	0.75	0.094	0.25	**16**	4	16	0.75	0.09	0.5	0.06	**≥32**	**≥256**	6
K. pneumoniae ALF301	Lettuce	23°32'03.8″ S; 46°44'08.4″ W	4	**≥256**	**≥32**	8	**24**	4	**48**	0.25	0.023	0.09	**≥1024**	4	3	0.12	0.04	0.06	0.03	**≥32**	**≥256**	**64**
K. pneumoniae RUC232	Arugula	23°13′48.0″ S; 46°11'24.0″ W	**≥256**	**≥256**	**≥32**	**16**	12	4	**16**	0.38	0.032	0.25	1	8	**≥32**	**16**	**4**	0.5	0.06	2	6	**16**
E. coli ESP110	Spinach	23°23′24.0″ S; 47°07'48.0″ W	**≥256**	**≥256**	**≥32**	**16**	4	12	**32**	0.25	0.094	0.19	**16**	8	16	1	0.04	0.25	0.03	**≥32**	**≥256**	**64**
E. coli REP215	Cabbage	23°25′12.0″ S; 47°15′00.0″ W	4	**≥256**	**≥32**	8	**16**	12	**16**	0.19	0.047	0.25	**48**	16	**≥256**	**≥32**	**≥32**	**≥32**	**≥32**	**≥32**	6	1.5
E. coli REP237	Cabbage	23°25′12.0″ S; 47°15′00.0″ W	**≥256**	**≥256**	**≥32**	12	**≥32**	3	**24**	0.38	0.032	0.19	0.5	4	6	0.5	0.06	0.25	0.12	**≥32**	3	**192**

a Geographical coordinates indicate origin of cultivated vegetables.

b Ptz, piperacillin-tazobactam; Cef, cephalothin; Cro, ceftriaxone; Caz, ceftazidime; Fep, cefepime; Fox, cefoxitin; Atm, aztreonam; Ipm, imipenem; Mem, meropenem; Etp, ertapenem; Gen, gentamicin; Ami, amikacin; Nal, nalidixic acid; Cip, ciprofloxacin; Enr, enrofloxacin; Lvx, levofloxacin, Mxf, moxifloxacin; Sxt, sulfamethoxazole/trimethoprim; Chl, chloramphenicol; Tet, tetracycline. Resistant MIC values are shown in boldface type, with resistance profiles determined using the CLSI 2020 guidelines (69). For enrofloxacin and moxifloxacin, resistance profiles were determined using CLSI 2018 (70) and EUCAST 2021 (https://www.eucast.org/) guidelines, respectively.

### Identification of global clones and phylogenomic analysis.

Endophytic ESBL-producing isolates belonged to different sequence types (ST). In this regard, E. coli ESP110, REP215, and REP237 from spinach and cabbage belonged to ST4012 and the international ST648 and ST38, respectively. K. pneumoniae ALF301 belonged to ST198 and K. pneumoniae RUC232 belonged to the new sequence type ST2739, a single-locus variant of international ST307. E. cloacae ESP151 isolated from spinach was assigned to the new ST927.

**FIG 1:**
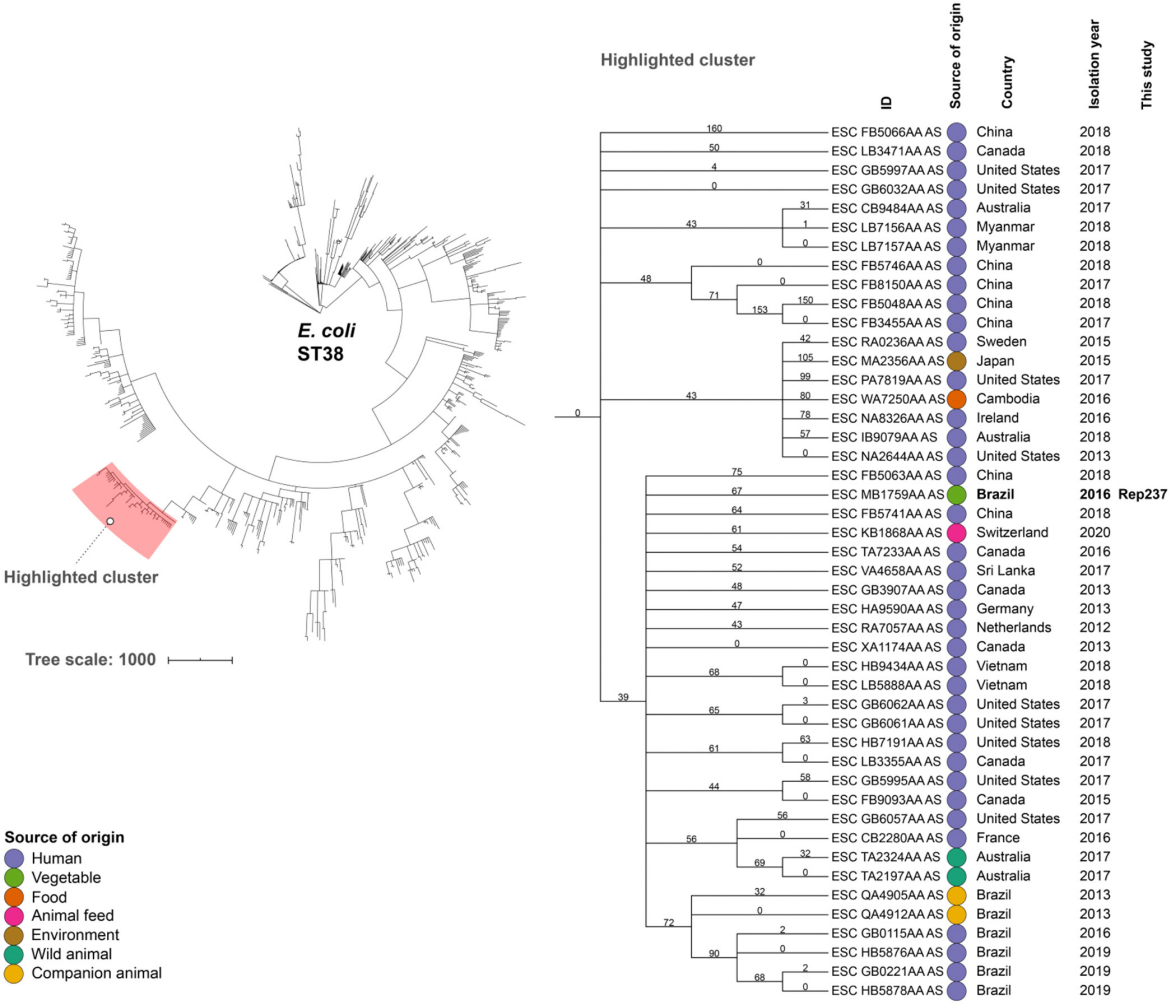
Phylogenomic analysis of a CTX-M-15-positive Escherichia coli REP237 strain, isolated from cabbage, in relation to an international collection of genomes of E. coli strains belonging to sequence type (ST) 38. On the left, the image shows a minimum spanning tree based on wgMLST of 799 worldwide distributed E. coli strains belonging to ST38, constructed by the MSTree V2 tool from EnteroBase. The E. coli strain REP237 was organized in the cluster highlighted in red. The highlighted cluster includes a partial depiction of the tree, including the Enterobase identification (ID), source of origin, country, and isolation year of genomically related isolates. The figure was generated with iTOL v.5.5 (https://itol.embl.de). An interactive version of the tree can be found at https://itol.embl.de/tree/14310712557248381595353218.

**FIG 2:**
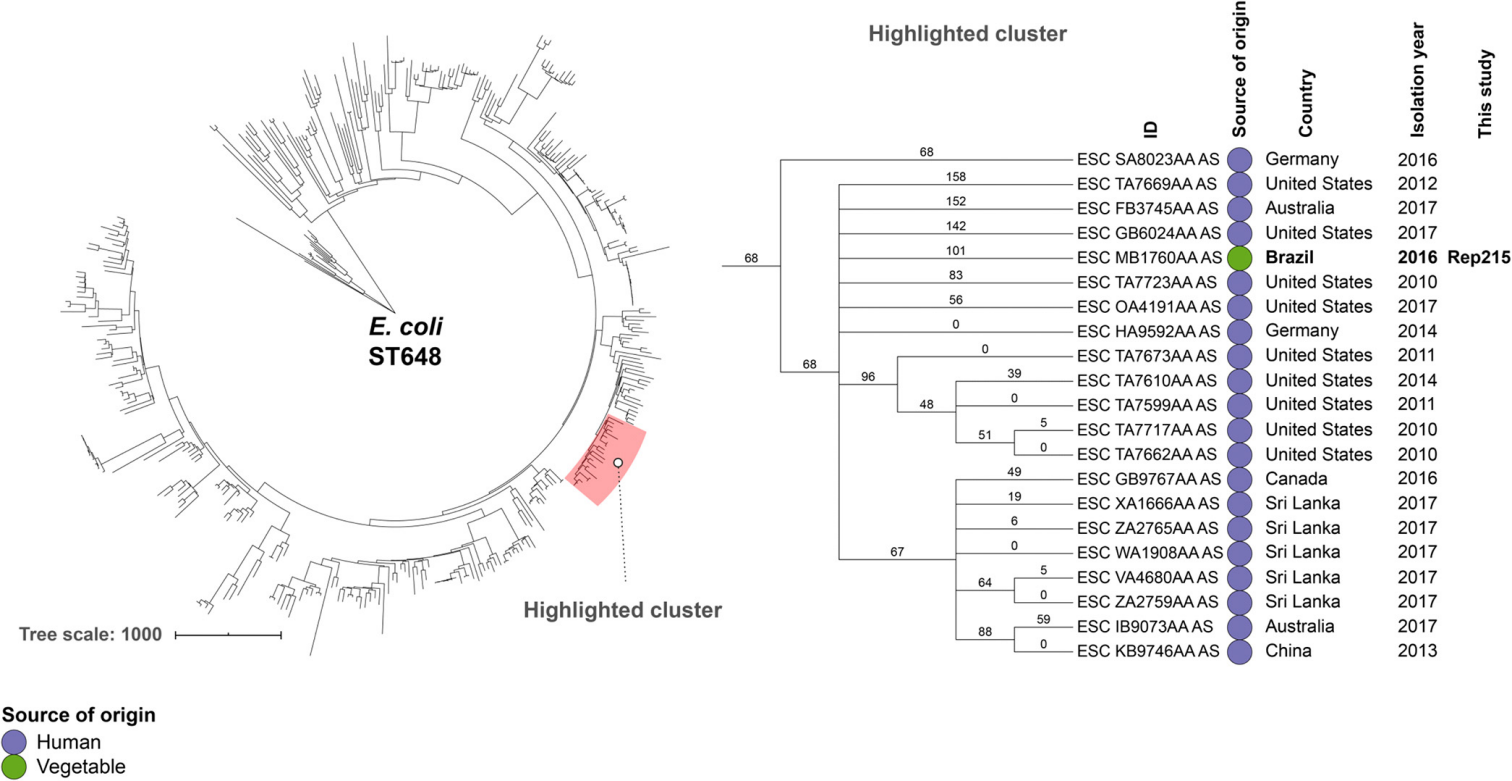
Phylogeny of a CTX-M-15-producing E. coli REP215 strain, isolated from cabbage, in relation to an international collection of genomes of E. coli strain belonging to ST648. On the left, the image shows a minimum spanning tree based on wgMLST of 390 worldwide distributed E. coli strains belonging to ST648, constructed by the MSTree V2 tool from EnteroBase. The E. coli strain REP215 was organized in the cluster highlighted in red. The highlighted cluster includes a partial depiction of the tree, including the Enterobase identification (ID), source of origin, country, and isolation year of genomically related isolates. The figure was generated with iTOL v.5.5 (https://itol.embl.de). An interactive version of the tree can be found at https://itol.embl.de/tree/14310712557248341595353217.

Genomic relatedness analysis of 798 assembled genomes of globally reported E. coli ST38 assigned E. coli strain REP237 to a cluster comprising human E. coli genomes from Asia, Europe, and America (including Brazilian human isolates), two wild animal E. coli genomes from Australia, one animal feed E. coli genome from Switzerland, and two companion animal genomes from Brazil (Fig. 1). On the other hand, the minimum spanning tree for E. coli REP215 isolate and the other 389 genome assemblies belonging to ST648 assigned E. coli REP215 to a cluster comprising human genomes from Europe, Asia, America, and Oceania (Fig. 2).

While genomes of E. cloacae and K. pneumoniae strains belonging to ST927 and ST2739, respectively, were not publicly available for comparative phylogenetic analysis, comparative core-genome multilocus sequence type (cgMLST) analysis of endophytic K. pneumoniae strain ALF301 with human K. pneumoniae strains belonging to ST198, previously identified in Brazil, revealed that endophytic K. pneumoniae ALF301 differs in 23 and 72 cgMLST alleles from human K. pneumoniae ICBKpBL-III-03(1) (GenBank acession number NIHK00000000.1) and ICBKpBL-III-02(1) (GenBank acession number: NGJM00000000.1) strains, respectively.

### Resistome, virulome, and identification of endophytic and acid tolerance genes.

**FIG 3:**
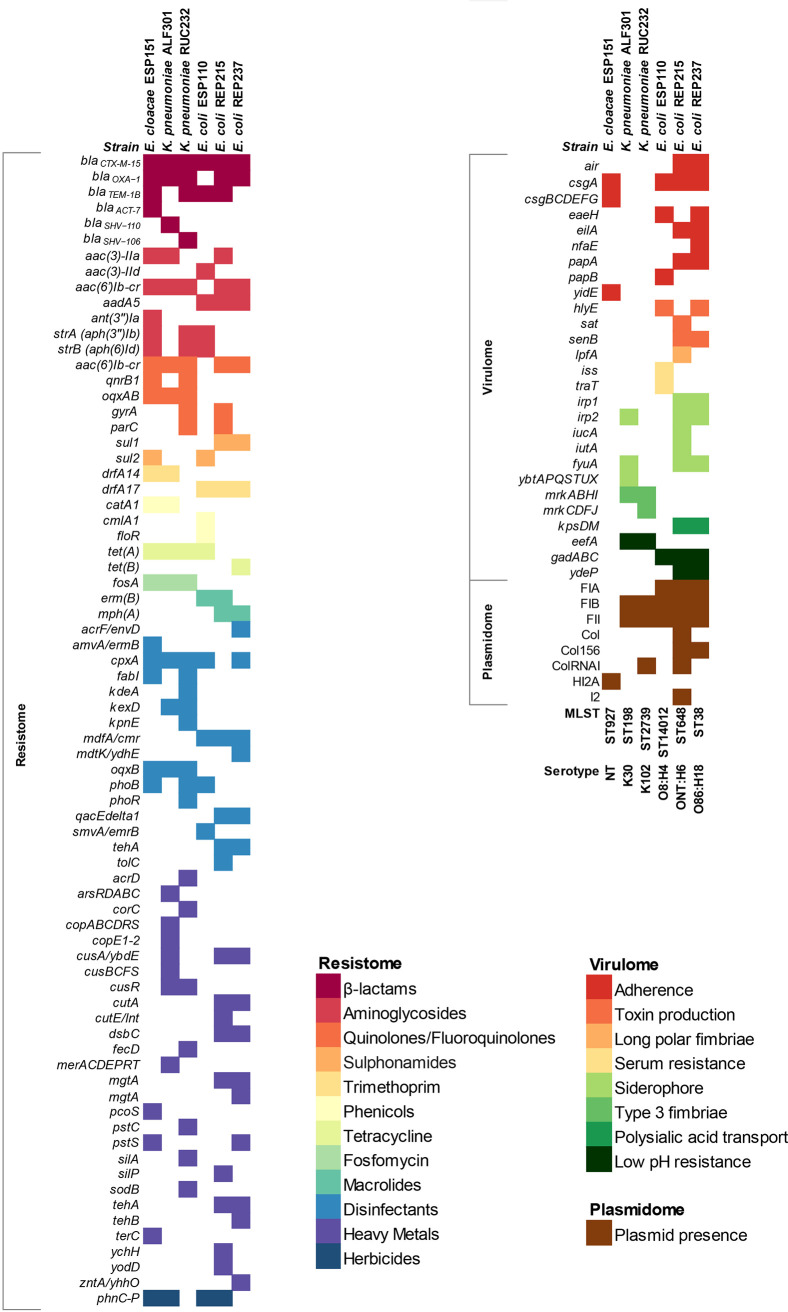
Heat map of resistome, virulome, plasmidome, and MLST and serotype data generated from whole-genome comparative genomics for endophytic multidrug-resistant *Enterobacterales* isolated from commercial vegetables. NT, not typed. For all predicted genes, a >90% identity threshold was used as a filter for identification.

Whole-genome sequence (WGS) analysis revealed that in all endophytic strains, ESBL production was associated with the presence of *bla*_CTX-M-15_ genes (Fig. 3). Additionally, the *bla*_OXA-1_ β-lactamase gene was further identified in all strains, except in E. coli ESP110. On the other hand, while E. coli strains ESP110 and REP215, K. pneumoniae RUC232, and E. cloacae ESP151 carried the *bla*_TEM-1B_ β-lactamase gene, both K. pneumoniae strains were also *bla*_SHV_-positive. In addition to beta-lactam resistance genes, the presence of resistance determinants to aminoglycosides (*strA*, *strB*, *aac*(*3*)*−II*, *aac*(*6’*)*Ib−cr*, *aadA5* and *ant(3″)Ia*), quinolones (*aac*(*6’*)*Ib−cr*, *qnrB1*, *oqxA*, and *oqxB*), sulfonamides (*sul1* and *sul2*), trimethoprim (*drfA14* and *drfA17*), phenicols (*catA1*, *cmlA1*, and *floR*), tetracyclines (*tetA* and *tetB*), fosfomycin (*fosA*) and macrolides (*ermB* and *mphA*) was confirmed among endophytic strains (Fig. 3). Substitutions Thr-83→Ile and Ser-80→Ile in the quinolone resistance-determining region (QRDR) of GyrA and ParC, respectively, were identified in the quinolone-resistant K. pneumoniae RUC232 strain, whereas substitutions Ser-83→Leu and Asp87→Asn in GyrA, and Ser-80→ Ile in ParC were found in E. coli REP215 (Fig. 3).

Heavy metals resistance gene clusters, such as *cusSRCFBA*, *copE2ABCDRSE1*, *arsRDABC*, and *merRTPCAD*, encoding resistance to copper/silver, copper, arsenic, and mercury, respectively, were identified in K. pneumoniae ALF301 isolated from commercial lettuce (Fig. 3), whereas E. coli REP237 carried tellurite resistance genes *tehA*/*tehB*. On the other hand, E. coli REP215, E. coli ESP110, K. pneumoniae ALF301, and E. cloacae ESP151 harbored the *phnC-P* gene system conferring resistance to glyphosate herbicide.

Regarding antiseptics and disinfectants, genes predicted to confer tolerance to hydrogen peroxide (*cpxA* and *kpnE*) and resistance to quaternary ammonium compounds (*acrF/envD*, *amvA/ermB*, *cpxA*, *kpnE*, *mdfA/cmr*, *mdtK/ydhE*, *oqxB*, *phoB*, *phoR*, *qacEdelta1*, *smvA/emrB*, *tehA*, and *tolC*), phenol (*tolC*), triclosan (*fabI*, *kpnE*, *oqxB*, and *tolC*), biguanides/chlorhexidine (*cpxA*, *kpnE*, *oqxB*, *phoB*, *phoR*, and *qacEdelta1*), organosulfate/sodium dodecyl sulfate compounds (*acrF/envD*, *amvA/ermB*, *kdeA*, *kpnE*, *oqxB*, *qacEdelta1*, and *tolC*), and/or ionic detergents/sodium deoxycholate (*kexD*, *kpnE*, *mdtK/ydhE*, and *qacEdelta1*) were also identified in all CTX-M-15-positive endophytic isolates (Fig. 3).

Virulome analysis of E. coli strains revealed the presence of genes involved in adherence (*air*, *eilA*, and *nfaE*), toxin production (*sat* and *senB*), long polar fimbriae (*lpfA*), increased serum survival (*iss*), and acid resistance (*gad*). In this regard, E. coli REP215 and REP237 belonged to the phylogroup D known for including highly virulent lineages, whereas E. coli ESP110 was assigned to the low-virulence phylogroup A, common among commensal lineages (18). Virulome analysis of K. pneumoniae strains confirmed genes encoding the production (*irp2* and *ybt*) and uptake (*fyuA*) of the siderophore yersiniabactin, and/or genes encoding type 3 fimbriae (*mrk* gene cluster) (Fig. 3). On the other hand, K. pneumoniae RUC232 showed an identical capsular polysaccharide serotype (KL102-*wzi*173) and O-locus (O2v2) than K. pneumoniae strains belonging to clonal complex CC307 (19), whereas K. pneumoniae ALF301 of ST198 displayed *ybt*16, ICEKp12, *wzi*85, KL30, and O1v1 serotype. In E. cloacae ESP151, genes involved in hyperadherence (*yidE*) and curli fimbriae formation (*csgABCDEFG* operon) were predicted (Fig. 3).

Genetic determinants contributing to an endophytic lifestyle, such as genes for nitrogen supply favoring plant growth (*narI*, *narJ*, and *nirB*), were found in all CTX-M-15-positive strains (20, 21). On the other hand, genes for biosynthesis of 2,3-butanediol, involved in plant growth (22), were found in all K. pneumoniae and E. cloacae strains. Furthermore, genes encoding chitinase ChiC (EC 3.2.1.14) were identified in E. cloacae ESP151, K. pneumoniae ALF301 and RUC232, and E. coli ESP110 strains. Genes encoding cellulase A 3 (EC 3.2.1.4) were harbored by all endophytic ESBL producers.

### Plasmidome, horizontal transfer of plasmids, and genetic environments of *bla*_CTX-M-15_ ESBL genes.

IncFIB and IncFII plasmid replicon types were harbored by CTX-M-15-producing endophytic strains. However, while in E. coli and K. pneumoniae strains the *bla*_CTX-M-15_ gene was carried on IncFIB plasmids, in E. cloacae this gene was harbored by an IncHI2A plasmid (Fig. 3). Conjugation assays confirmed transfer of *bla*_CTX-M-15_/IncFIB plasmids from E. coli strains REP215 (ST648), REP237 (ST38), and ESP110 (ST4012) at frequencies of 8.67 × 10^−4^, 2.33 × 10^−3^, and 5.14 × 10^−3^ transconjugants/recipient cell, respectively. For K. pneumoniae RUC232 and ALF301, and E. cloacae ESP151, transfer of plasmid carrying the *bla*_CTX-M-15_ gene was only achieved by transformation with efficiency of 4.95 × 10^5^, 6.12 × 10^5^, and 1.05 × 10^6^ transformants/μg of plasmid, respectively.

**FIG 4:**
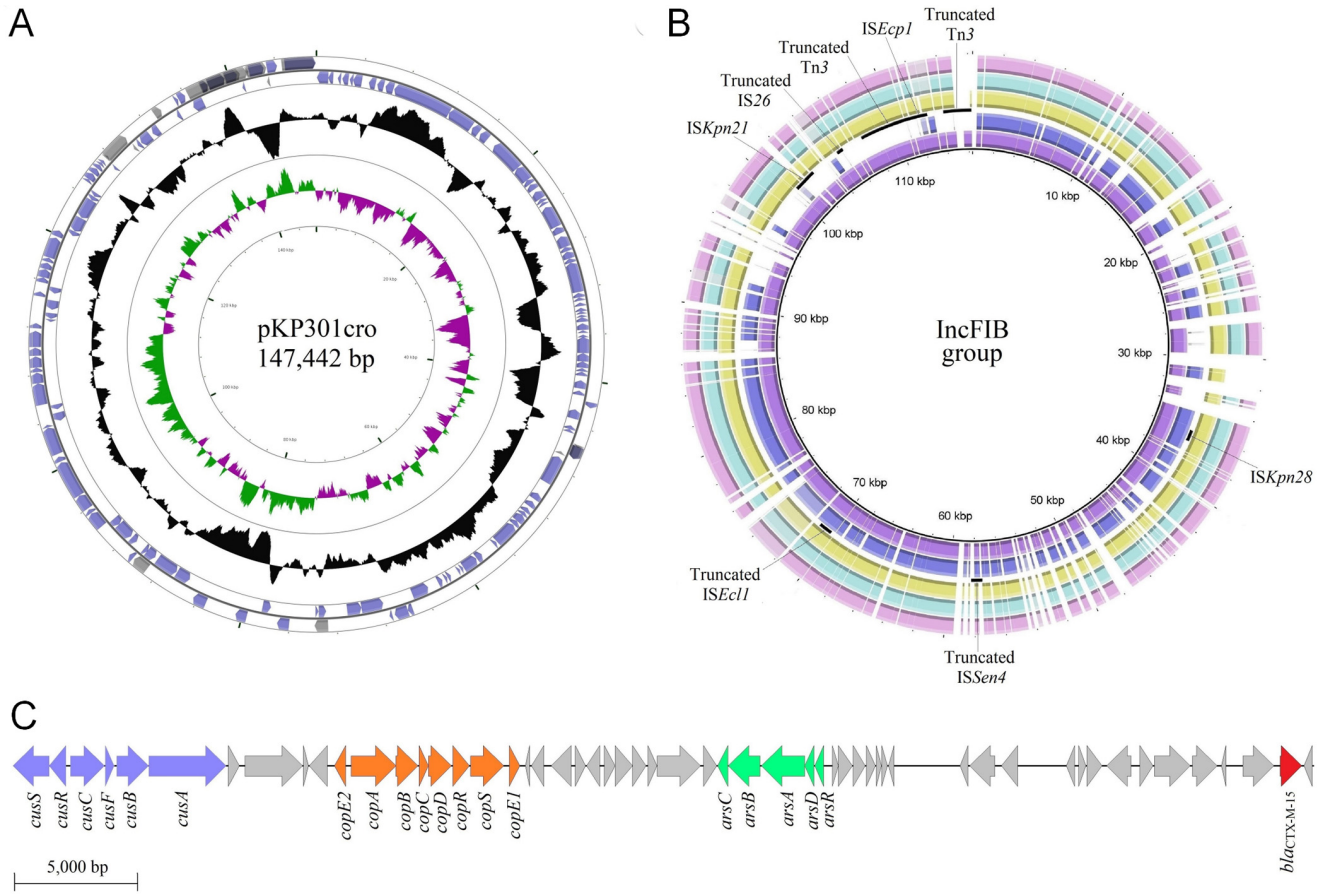
(A) Overview of the pKP301cro plasmid harbored by the endophytic K. pneumoniae ALF301 ST198 isolated from lettuce. Blue, protein coding sequences; gray, transposable elements; black, GC content; green, positive GC skew; purple, negative GC skew. (B) Comparison of IncFIB plasmids carrying β-lactamase genes. Low identities (70% to 50%) between plasmid proteins are indicated as lighter shades. Matches with less than 50% identity and no matches appear as blank spaces. In pink, pLGP4 plasmid (GenBank: MF116002.1) from uncultured bacterium; in green, p6234-198.371kb plasmid (GenBank: CP010390.1); in yellow, pKPSH11 plasmid (GenBank: KT896504.1); in blue, pKP301cro plasmid (GenBank: KY495890.1); and in purple, pKPN3-307_typeD plasmid (GenBank: KY271407.1) of K. pneumoniae strains from bodily fluid, wastewater, lettuce, and aspirate bronchial, respectively. Black arcs indicate transposable elements in pKP301cro. (C) Region from pKP301cro showing resistance genes to silver/copper (blue, *cusSRCFBA*), copper (orange, *copE2ABCDRSE1*), arsenic (green, *arsRDABC*), and β-lactams (red, *bla*_CTX-M-15_).

In K. pneumoniae ALF301, the *bla*_CTX-M-15_ gene was carried on an IncFIB plasmid named pKP301cro. The pKP301cro plasmid is 147,442 bp in size, with G+C content of 50.78%, coharboring *cusSRCFBA*, *copE2ABCDRSE1*, and *arsRDABC* gene clusters (Fig. 4A and C). Interestingly, this plasmid showed significant divergence from others of the same incompatibility group identified in clinical and environmental strains (Fig. 4B).

**FIG 5:**
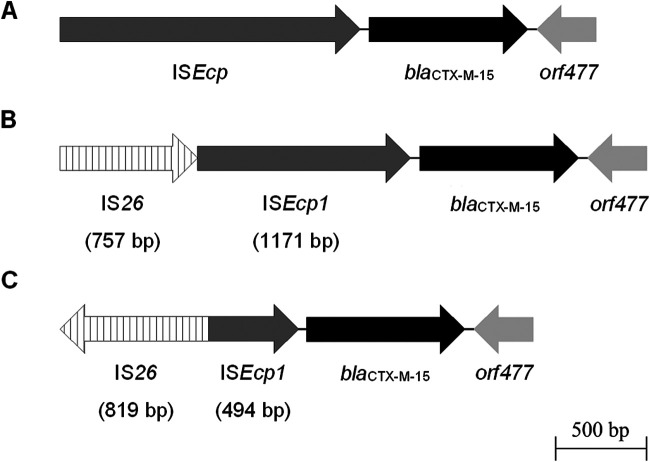
Schematic representation of genetic environments surrounding *bla*_CTX-M-15_ in endophytic *Enterobacterales*. (A) International *bla*_CTX-M-15_ genetic environment (23), identified in endophytic E. coli of ST648 (REP215, GenBank accession number: MG844172), K. pneumoniae of ST198 and ST2739 (ALF301, GenBank accession number: MG844168, and RUC232, GenBank accession number: MG844173, respectively), and E. cloacae (ESP151, GenBank accession number: MG844171) strains. (B) The novel environment identified in E. coli REP237 of ST38 (GenBank accession number: MG844170) presents a 1,171-bp IS*Ecp1* truncated by an incomplete IS*26* upstream of the *bla*_CTX-M-15_ gene. (C) The novel environment identified in E. coli ESP110 of ST4012 (GenBank accession number: MG844169) presents a 494-bp IS*Ecp1* truncated by an inverted IS*26*.

Three different genetic environments were found surrounding *bla*_CTX-M-15_ (Fig. 5). In K. pneumoniae and E. cloacae strains and one E. coli strain (REP215), the international *bla*_CTX-M-15_ genetic environment was confirmed (Fig. 5A) (23). Moreover, two novel environments were present in the endophytic E. coli strains belonging to ST38 (REP237) and ST4012 (ESP110) isolated from cabbage and spinach, respectively (Fig. 5B and C). In these novel environments, the ST38 lineage showed an 1,171-bp IS*Ecp1* insertion element truncated by an incomplete IS*26* upstream of the *bla*_CTX-M-15_ gene, while the ST4012 lineage exhibited a 494-bp IS*Ecp1* truncated by an inverted IS*26*.

### Endophytic properties of CTX-M-15-producing *Enterobacterales*.

This assay was designed to exclusively evaluate endophytic properties and plant-colonizing abilities of ESBL-producing isolates by using a common bean (Phaseolus vulgaris) model, determining endophytic bacterial loads recovered from root and shoot tissues (leaves) after inoculation of sterile sprouts obtained from surface-sterilized bean seeds (Table 2). In this regard, all CTX-M-15-positive isolates were able to endophytically colonize common bean seedlings. In order to evaluate endophytic properties and plant-colonizing abilities, bacterial burdens were evaluated at 15 days after inoculation of common bean. All strains efficiently colonized the interior of the root and shoot systems, supporting endophytic behaviors. Significantly higher bacterial counts in the root (*P < *0.05) were determined for CTX-M-15-producing K. pneumoniae ALF301 and RUC232. Otherwise, the E. cloacae ESP151 displayed a higher bacterial burden within the shoot than the other strains (*P* < 0.05) (Table 2).

**TABLE 2:** Endophytic bacterial load in root and shoot tissues of common bean (Phaseolus vulgaris) inoculated with CTX-M-15-producing *Enterobacterales* isolated from commercial vegetables

Inoculating strain^*d*^	CFU/g tissue ± SD	
	Root	Shoot
K. pneumoniae ALF301^*a*^	1.6 ± 0.1 × 10^5^	6.9 ± 0.4 × 10^2^
K. pneumoniae RUC232^*a*^	8.7 ± 0.5 × 10^4^	3.8 ± 0.3 × 10^2^
E. cloacae ESP151^*b*^	6.4 ± 0.4 × 10^4^	2.1 ± 0.1 × 10^3^
E. coli REP215	3.9 ± 0.4 × 10^4^	7.0 ± 0.9 × 10^1^
E. coli ESP110	3.2 ± 0.6 × 10^4^	3.1 ± 0.5 × 10^2^
E. coli REP237	5.1 ± 0.5 × 10^3^	7.5 ± 1.0 × 10^1^
A. baumannii ATCC 19606	-^*c*^	-^*c*^

a Significantly higher bacterial counts in the root (*P *< 0.05).

b Significantly higher bacterial counts in the shoot (*P *< 0.05).

c Undetectable bacterial colonies.

d Common beans (Phaseolus vulgaris) grains were surface-sterilized and, after a two-day germination, sprouts were incubated for 30 min with bacterial suspension (OD_600_ = 1.5) and transferred to plant culture bottles with Murashige and Skoog medium. Endophytic bacterial load in root and shoot tissue was assessed at 15 days after inoculation. All assays were performed in triplicate. Acinetobacter baumannii ATCC 19606 was used as a negative control for endophytic colonization.

### Tolerance of CTX-M-15-producing endophytic *Enterobacterales* to acid pH.

Initially, all *Enterobacterales* strains were grown in Trypticase soy broth (TSB) medium at pH 7.0 to a cell density of ∼1 × 10^8^ CFU/ml, and the cells were collected, washed, and transferred into the same medium at pH ranging from 7.0 to 2.0, at a final concentration of 10^5^ cells per well. For all strains, no reduction of CFU/ml was observed after 24 h of incubation at pH 6.0 to 5.0. However, at pH 4.0, while no reduction in CFU/ml of K. pneumoniae was observed after 24 h of incubation, for E. coli and E. cloacae the cell density was reduced by 2 to 3 log CFU/ml. On the other hand, pH 3.0 led to reduction of K. pneumoniae and E. coli cell densities by 3 to 5 and 2 to 4 log CFU/ml at 1 h and 2 h of incubation, respectively, whereas for all strains CFU/ml were undetectable after 24 h of incubation. Finally, only E. coli strains presented tolerance to pH 2.0, where cell density was reduced by 3 to 4 and 2 to 3 log CFU/ml at 1 h and 2 h of incubation, respectively.

## DISCUSSION

Members of the *Enterobacterales* order have been shown to colonize and benefit plant growth in various crops, such as wheat, maize, rice, and cucumber (4, 24–27). Worryingly, ESBL-producing *Enterobacterales*, including CTX-M-15 producers, have also been reported in vegetables, representing a risk of human exposure to MDR critical priority pathogens through this food source (7, 13, 14). In this regard, the occurrence of epiphytic ESBL producers in fresh vegetables has been described in North American, Asian, and European countries (13, 28). In South America, ESBL-positive E. coli of types ST44 and ST410 have been recently identified in fresh vegetables sold in Ecuador (14).

In Brazil, the largest and most populated country in South America, ESBL production has been documented to be more challenging than in developed countries. In fact, ESBL-producing *Enterobacterales* are endemic in both hospital and community settings (29–31). K. pneumoniae and E. coli have been frequently associated with the production of CTX-M-15 ESBLs (32). Worryingly, in this country, CTX-M-15 producers have also been identified in chicken meat, wild and food-producing animals, pets, Amazonian fish, and aquatic environment samples (11, 33–44), whereas its presence in vegetables has not been investigated in deep, so far.

Most studies conducted to evaluate contamination of commercial vegetables by MDR pathogens have focused on epiphytic bacteria, which colonize the surface of leaves, roots, seeds, and fruits (5) and thus remain susceptible to disinfecting methods, which have been shown to be effective against bacteria colonizing fruit and vegetable surfaces (45). Therefore, identification of CTX-M-15-producing *Enterobacterales* with endophytic lifestyles is a critical public health issue, since endophytic bacteria colonize protected sites of internal plant tissues, from where they are able to resist conventional treatments used for disinfecting leafy vegetables (13, 46). Consequently, CTX-M-15 producers with endophytic lifestyles could begin to colonize hosts that use vegetables in the diet.

To support this hypothesis, we performed assays measuring tolerance to acid pH using TSB adjusted to pH 2, 3, 4, 5, 6, and 7 to define the survival of the endophytic CTX-M-positive *Enterobacterales* identified in this study. Low pH values were chosen in order to evaluate the ability to survive transit through the acidic conditions of the stomach, which is essential for successful colonization of the mammalian host by commensal and pathogenic bacteria (47). Interestingly, both K. pneumoniae and E. coli strains exhibited tolerance to acid pH, which was supported by the presence of *eefA* and *gad* genes. While the *gad* system helps to maintain a near-neutral intracellular pH when cells are exposed to extremely acidic conditions, *eefA* confers to the bacteria an acid tolerance response to inorganic acids (48).

Although, the origins of clinically relevant CTX-M-15-producing bacteria found in fresh vegetables in this study were not investigated, they could originate from human (as sewage), animal (manure and wild animal feces), and/or environmental (such as contaminated soil and irrigation water) sources that come into contact with crops (49–52). In this regard, colonization of vegetables can occur through entrances such as stomata, lenticels, root hairs, lesions, and emergent surfaces of radicle and lateral roots (53, 54). Additionally, animal and human pathogens, especially E. coli pathotypes, are also able to colonize endospheres (4). This last hypothesis could be supported by the endophytic properties and plant-colonizing abilities of CTX-M-15-producing strains, as observed in this study. In fact, using the common bean model, all strains exhibited endophytic colonization ability.

Another clinically relevant result of this study is the identification of endophytic CTX-M-15-producing E. coli strains belonging to pandemic high-risk clonal complexes CC38 and CC648, and K. pneumoniae of complex CC307, which have been associated with extraintestinal diseases and (mainly) bloodstream and urinary tract infections (BSIs and UTIs, respectively) (19, 55–58). In Brazil, these international clones have been previously identified in human and animal infections and in polluted environments, denoting One Health implications (38, 59–63).

The wide host range of these critical priority clonal complexes, including different vegetables evaluated in this study, supports a genetic versatility and adaptation mediated by the gene content, which includes genes conferring endophytic properties and resistance to antibiotics, biocides, and heavy metals. In fact, an IncFIB plasmid (pKP301cro) coharboring the *bla*_CTX-M-15_ gene and heavy metals resistance genes (i.e., *cusSRCFBA*, *copE2ABCDRSE1*, and *arsRDABC*) was identified. Heavy metals can come from contaminated soil, irrigation water, and inorganic fertilizers and pesticides commonly used in agricultural practices, which remain in the environment for long periods, accumulating in leaves, stem, and root of plants (45, 64–67). Consequently, these compounds, as well as biocides, may act as selectors of strains resistant to antibiotics. Therefore, the presence of multidrug-resistant pathogens displaying endophytic lifestyles and broad resistomes, resident in fresh vegetables, denotes environmental and food contamination mediated by anthropogenic activities. Future studies that include the analysis of a higher number of vegetables samples of different origins and the quantitative analysis of ESBL producers are worthy of further investigation, in order to gather data for risk assessment.

In conclusion, the occurrence of international clones of critical World Health Organization priority pathogens that are producing CTX-M-15 ESBL, harboring a broad resistome, and displaying endophytic lifestyles in fresh vegetables is a public and environmental health problem; it denotes contamination mediated by anthropogenic activities and a potential risk of human and animal exposure to antibiotic-resistant bacteria and/or their resistance genes. Therefore, fresh vegetables marketed for consumption can act as a figurative Trojan horse for the hidden spread of multidrug-resistant and ESBL-producing pathogens, which could be important bioindicators of environmental and food contamination.

## MATERIALS AND METHODS

### Isolation of endophytic bacteria displaying a broad-spectrum cephalosporin-resistant profile from surface-sterilized fresh vegetables.

During a Brazilian surveillance study (OneBR project), conducted to characterize the burden of antimicrobial resistance associated with critical WHO priority pathogens, 48 samples of fresh vegetables collected from the São Paulo State Food Supply Company, the largest supply center in South America, were investigated. Vegetables included lettuce (*n =* 6), spinach (*n =* 6), escarole (*n =* 6), watercress (*n =* 4), beet (*n =* 4), arugula (*n =* 4), kale (*n =* 4), radish (*n =* 4), cabbage (*n =* 4), celery (*n =* 2), leek (*n =* 2), and chicory (*n =* 2). All samples collected were immediately stored in sealed plastic bags at 4°C and processed within 24 h. Samples were washed in running water and sanitized before the isolation of endophytic bacteria. For surface sterilization, ∼4 g of leaves were immersed sequentially in 70% ethanol (1 min), sodium hypochlorite (2.5% chlorine, 4 min), and 70% ethanol (30 s), and then washed three times in sterile distilled water (68). Aliquots of the sterile water used in the final rinse were plated directly onto nutrient agar to confirm the sterilization protocol. For the isolation of ESBL-positive endophytic bacteria, surface-sterilized leaves were macerated in 12 ml of saline solution, serially diluted, and plated in triplicate on MacConkey agar supplemented with ceftriaxone (2 μg/ml). After 24 h of incubation at 37°C, colonies were picked from the selective plates, subcultured, and streaked to obtain pure cultures. Identification of isolates was performed using the Vitek 2 system (bioMérieux, Marcy l’Etoile, France).

### Antimicrobial susceptibility testing and phenotypic confirmation of ESBLs.

Bacterial isolates were subjected to antimicrobial susceptibility testing by the disk diffusion method, whereas MICs were determined by Etest strips (bioMérieux, Marcy l’Etoile, France), with interpretative criteria according to CLSI (69, 70) or EUCAST (www.eucast.org). ESBL production was screened by the double-disk synergy test (71), with further confirmation by using Etest ESBL strips containing ceftazidime alone and in combination with clavulanic acid (bioMérieux, Marcy l’Etoile, France).

### Whole-genome sequencing and bioinformatic analysis.

All ESBL-producing endophytic isolates underwent whole-genome sequencing (WGS). For genome sequencing, total DNA was extracted from overnight cultures using the PureLink genomic DNA minikit (Thermo Fisher Scientific, USA) according to the manufacturer's instructions. Sequencing was performed using the MiSeq platform (Illumina, San Diego, CA) (300 bp paired-end) and the reads were *de novo* assembled using SPAdes v.3.9 and A5-Miseq pipeline (72, 73). Sequence types (STs), serotypes, plasmid replicon types, antimicrobial resistance genes, and virulence genes were identified using MLST 2.0, SerotypeFinder 2.0, PlasmidFinder 2.1, ResFinder 4.1, and VirulenceFinder 2.0, respectively, available from the Center for Genomic Epidemiology (https://cge.cbs.dtu.dk/services/), and databases for bacterial genotyping from the Pasteur Institute (https://bigsdb.pasteur.fr/). Resistance genes with uncertain assignment by ResFinder were checked manually and further blasted in NCBI. Analysis of transposable elements flanking *bla*_CTX-M_ genes was performed with ISfinder (74). For E. coli, virulence phylogroups were detected using the online Clermont typing tool (http://clermontyping.iame-research.center/). K. pneumoniae were further analyzed using Kleborate (https://github.com/katholt/Kleborate) to screen assemblies to confirm the species designation, multilocus sequence type (MLST), antibiotic-resistance genes, ICE*Kp*-associated virulence loci (yersiniabactin [*ybt*] and colibactin [*clb*]), and K (capsule) and O antigen (LPS) serotypes (75–77). Biocide-, heavy metal-, and disinfectant-resistance genes, along with genes to withstand acidic conditions, were identified using the BacMet-Scan script (http://bacmet.biomedicine.gu.se/) against the experimentally confirmed database v.2.0, using an E value = 1 and a threshold of >90% of identity and coverage.

Comparative phylogeny analysis of publicly available genomes of E. coli ST38 and ST648, from different countries, was performed using a minimum spanning tree constructed in Enterobase using the MSTree V2 algorithm and the wgMLST scheme (https://enterobase.warwick.ac.uk/species/index/ecoli), which consists of 25,002 pangenome genes present in E. coli genomes, representing most of the diversity in Enterobase at the time (February 2021) (https://enterobase.readthedocs.io/en/latest/pipelines/escherichia-statistics.html). Images were generated with iTOL v.5.5 (https://itol.embl.de). Comparative phylogeny of publicly available genomes of K. pneumoniae belonging to ST198 was performed using core-genome MLST (cgMLST) analysis and the BacWGSTdb database (http://bacdb.cn/BacWGSTdb/).

### Conjugation and transformation of plasmids carrying ESBL genes.

E. coli C600 (Str^R^) and E. coli J53 (Az^R^) were used as recipient strains in mating experiments with endophytic ESBL-producing E. coli strains as donors, in the ratio 3:1 (recipient:donor) in LB broth. Transconjugants were selected using MacConkey agar supplemented with ceftriaxone (2 μg/ml) and streptomycin (2,000 μg/ml), or ceftriaxone (2 μg/ml) and sodium azide (200 μg/ml). For transformation assay, plasmids were extracted by the alkaline lysis method (78) and ultracompetent E. coli TOP10 was heat shock transformed, as previously described (79), increasing the thermal shock time at 42°C to 1.5 min. Positive transconjugants and transformants were confirmed by ESBL production, as described above.

### Endophytic properties of ESBL-producing *Enterobacterales*.

Endophytic properties of ESBL-producing isolates were evaluated using a common bean (Phaseolus vulgaris) model (68), with modifications. In brief, bean seeds were surface sterilized and then incubated at 30°C until the early growth of the radicle (80). After two days of germination, sprouts were immersed for 30 min in the bacterial cell suspension (optical density at 600 nm [OD_600_] = 1.5) and transferred to plant culture bottles with Murashige and Skoog medium, which were then incubated at 30°C for 15 days. Thereafter, bean plants were aseptically excised into root and shoot and endophytic bacteria were isolated from each of them, as described above. The recovered isolates were confirmed by detecting ESBL genes and by assessment of the clonal relatedness with the strains used to inoculate the sprouts, as determined by comparative enterobacterial repetitive intergenic consensus (ERIC)-PCR analysis (81). All assays were performed in triplicate. Acinetobacter baumannii ATCC 19606 and sterile distilled water were used as negative controls.

### Tolerance of endophytic ESBL (CTX-M-15)-producing *Enterobacterales* to acid pH.

Trypticase soy broth (TSB) culture medium was prepared to cover acid pH scales ranging from 2.0 to 7.0. Volumes of 50 ml of TSB were adjusted individually to a final pH of 2.0, 3.0, 4.0, 5.0, 6.0, and 7.0 by aseptically adding 1 N HCl, and using a pH meter (82). Broths were sterilized and the pH was confirmed. Next, 200 μl of each broth at the different pH values were added per well in a 96-well flat-bottomed microtiter plate. All endophytic ESBL producers were tested for pH tolerance (83). In brief, each well of the microtiter plate was inoculated with bacterial cell suspension to a final concentration of 10^5^ cells per well and then the microplates were incubated at 35°C. After 1, 2, and 24 h of incubation, an aliquot (50 μl) of cell suspension was taken from each well, diluted 1:10, 1:100, 1:1,000, and 1:10,000, and cell viability was determined by plating 50 μl of each dilution on Trypticase soy agar (TSA) plates and incubating for 24 h at 35°C (84). All assays were performed in duplicate.

### Statistical analysis.

Data were subjected to analysis of variance (ANOVA) followed by the Duncan’s multiple range test with a significance level of *P < *0.05, using IBM SPSS Statistics 24 software (IBM, United States).

### Data availability.

Nucleotide sequences of endophytic CTX-M-producing *Enterobacterales* have been deposited in the GenBank database under accession numbers: PPHP01000000 (E. cloacae ESP151); MRWC01000000 (K. pneumoniae ALF301); PPHO01000000 (K. pneumoniae RUC232); PPHN01000000 (E. coli ESP110); PPHM01000000 (E. coli REP215); PPHL01000000 (E. coli REP237); KY354306.1 (plasmid pKP301b from K. pneumoniae ALF301); and KY495890.1 (plasmid pKP301cro from K. pneumoniae ALF301). Additionally, genomic data of E. coli ESP110, REP215, and REP237 and K. pneumoniae ALF301 and RUC232 strains are available on the OneBR platform (http://onehealthbr.com/) under numbers ID ONE110, ONE111, ONE112, ONE249, and ONE250, respectively.

## References

[1] Tacconelli E, Carrara E, Savoldi A, Harbarth S, Mendelson M, Monnet DL, Pulcini C, Kahlmeter G, Kluytmans J, Carmeli Y, Ouellette M, Outterson K, Patel J, Cavaleri M, Cox EM, Houchens CR, Grayson ML, Hansen P, Singh N, Theuretzbacher U, Magrini N, WHO Pathogens Priority List Working Group. 2018. Discovery, research, and development of new antibiotics: the WHO priority list of antibiotic-resistant bacteria and tuberculosis. Lancet Infect Dis 18:318–327. doi:10.1016/S1473-3099(17)30753-3. 29276051

[2] Bevan ER, Jones AM, Hawkey PM. 2017. Global epidemiology of CTX-M β-lactamases: temporal and geographical shifts in genotype. J Antimicrob Chemother 72:2145–2155. doi:10.1093/jac/dkx146. 28541467

[3] Humeniuk C, Arlet G, Gautier V, Grimont P, Labia R, Philippon A. 2002. β-Lactamases of *Kluyvera ascorbata*, probable progenitors of some plasmid-encoded CTX-M types. Antimicrob Agents Chemother 46:3045–3049. doi:10.1128/aac.46.9.3045-3049.2002. 12183268 PMC127423

[4] Hardoim PR, van Overbeek LS, Berg G, Pirttilä AM, Compant S, Campisano A, Döring M, Sessitsch A. 2015. The hidden world within plants: ecological and evolutionary considerations for defining functioning of microbial endophytes. Microbiol Mol Biol Rev 79:293–320. doi:10.1128/MMBR.00050-14. 26136581 PMC4488371

[5] Kumar J, Singh D, Ghosh P, Kumar A. 2017. Endophytic and epiphytic modes of microbial interactions and benefits, p 227–253. In Singh D, Singh H, Prabha R (ed), Plant-microbe interactions in agro-ecological perspectives. Springer Nature Singapore, Singapore,

[6] Hölzel CS, Tetens JL, Schwaiger K. 2018. Unraveling the role of vegetables in spreading antimicrobial-resistant bacteria: a need for quantitative risk assessment. Foodborne Pathog Dis 15:671–688. doi:10.1089/fpd.2018.2501. 30444697 PMC6247988

[7] O'Flaherty E, Solimini AG, Pantanella F, De Giusti M, Cummins E. 2019. Human exposure to antibiotic resistant-*Escherichia coli* through irrigated lettuce. Environ Int 122:270–280. doi:10.1016/j.envint.2018.11.022. 30449627

[8] Woerther PL, Burdet C, Chachaty E, Andremont A. 2013. Trends in human fecal carriage of extended-spectrum β-lactamases in the community: toward the globalization of CTX-M. Clin Microbiol Rev 26:744–758. doi:10.1128/CMR.00023-13. 24092853 PMC3811232

[9] Pitout JD. 2013. Enterobacteriaceae that produce extended-spectrum β-lactamases and AmpC β-lactamases in the community: the tip of the iceberg? Curr Pharm Des 19:257–263. doi:10.2174/13816128130207. 22934977

[10] Chong Y, Shimoda S, Shimono N. 2018. Current epidemiology, genetic evolution and clinical impact of extended-spectrum beta-lactamase-producing *Escherichia coli* and *Klebsiella pneumoniae*. Infect Genet Evol 61:185–188. doi:10.1016/j.meegid.2018.04.005. 29626676

[11] Melo LC, Oresco C, Leigue L, Netto HM, Melville PA, Benites NR, Saras E, Haenni M, Lincopan N, Madec JY. 2018. Prevalence and molecular features of ESBL/pAmpC-producing Enterobacteriaceae in healthy and diseased companion animals in Brazil. Vet Microbiol 221:59–66. doi:10.1016/j.vetmic.2018.05.017. 29981709

[12] Fuentes-Castillo D, Esposito F, Cardoso B, Dalazen G, Moura Q, Fuga B, Fontana H, Cerdeira L, Dropa M, Rottmann J, González-Acuña D, Catão-Dias JL, Lincopan N. 2020. Genomic data reveal international lineages of critical priority *Escherichia coli* harbouring wide resistome in Andean condors (*Vultur gryphus Linnaeus*, 1758). Mol Ecol 29:1919–1935. doi:10.1111/mec.15455. 32335957

[13] Reuland EA, Al Naiemi N, Raadsen SA, Savelkoul PH, Kluytmans JA, Vandenbroucke-Grauls CM. 2014. Prevalence of ESBL-producing Enterobacteriaceae in raw vegetables. Eur J Clin Microbiol Infect Dis 33:1843–1846. doi:10.1007/s10096-014-2142-7. 24848131 PMC4182617

[14] Ortega-Paredes D, Barba P, Mena-López S, Espinel N, Zurita J. 2018. *Escherichia coli* hyperepidemic clone ST410-A harboring *bla*_CTX-M-15_ isolated from fresh vegetables in a municipal market in Quito-Ecuador. Int J Food Microbiol 280:41–45. doi:10.1016/j.ijfoodmicro.2018.04.037. 29777948

[15] Campos J, Mourão J, Pestana N, Peixe L, Novais C, Antunes P. 2013. Microbiological quality of ready-to-eat salads: an underestimated vehicle of bacteria and clinically relevant antibiotic resistance genes. Int J Food Microbiol 166:464–470. doi:10.1016/j.ijfoodmicro.2013.08.005. 24036261

[16] Maeusli M, Lee B, Miller S, Reyna Z, Lu P, Yan J, Ulhaq A, Skandalis N, Spellberg B, Luna B. 2020. Horizontal gene transfer of antibiotic resistance from *Acinetobacter baylyi* to *Escherichia coli* on lettuce and subsequent antibiotic resistance transmission to the gut microbiome. mSphere 5:e00329-20. doi:10.1128/mSphere.00329-20. 32461272 PMC7253597

[17] Magiorakos AP, Srinivasan A, Carey RB, Carmeli Y, Falagas ME, Giske CG, Harbarth S, Hindler JF, Kahlmeter G, Olsson-Liljequist B, Paterson DL, Rice LB, Stelling J, Struelens MJ, Vatopoulos A, Weber JT, Monnet DL. 2012. Multidrug-resistant, extensively drug-resistant and pandrug-resistant bacteria: an international expert proposal for interim standard definitions for acquired resistance. Clin Microbiol Infect 18:268–281. doi:10.1111/j.1469-0691.2011.03570.x. 21793988

[18] Clermont O, Christenson JK, Denamur E, Gordon DM. 2013. The Clermont *Escherichia coli* phylo-typing method revisited: improvement of specificity and detection of new phylo-groups. Environ Microbiol Rep 5:58–65. doi:10.1111/1758-2229.12019. 23757131

[19] Wyres KL, Hawkey J, Hetland MAK, Fostervold A, Wick RR, Judd LM, Hamidian M, Howden BP, Löhr IH, Holt KE. 2019. Emergence and rapid global dissemination of CTX-M-15-associated *Klebsiella pneumoniae* strain ST307. J Antimicrob Chemother 74:577–581. doi:10.1093/jac/dky492. 30517666 PMC6376852

[20] Lin JT, Stewart V. 1998. Nitrate assimilation by bacteria. Adv Microb Physiol 39:1–30. doi:10.1016/s0065-2911(08)60014-4. 9328645

[21] Kraiser T, Gras DE, Gutiérrez AG, González B, Gutiérrez RA. 2011. A holistic view of nitrogen acquisition in plants. J Exp Bot 62:1455–1466. doi:10.1093/jxb/erq425. 21239377 PMC3137434

[22] Liu W, Wang Q, Hou J, Tu C, Luo Y, Christie P. 2016. Whole genome analysis of halotolerant and alkalotolerant plant growth-promoting rhizobacterium *Klebsiella* sp. D5A. Sci Rep 6:26710. doi:10.1038/srep26710. 27216548 PMC4877636

[23] Dhanji H, Patel R, Wall R, Doumith M, Patel B, Hope R, Livermore DM, Woodford N. 2011. Variation in the genetic environments of *bla*_CTX-M-15_ in *Escherichia coli* from the faeces of travellers returning to the United Kingdom. J Antimicrob Chemother 66:1005–1012. doi:10.1093/jac/dkr041. 21393166

[24] Chelius MK, Triplett EW. 2000. Immunolocalization of dinitrogenase reductase produced by *Klebsiella pneumoniae* in association with *Zea mays* L. Appl Environ Microbiol 66:783–877. doi:10.1128/aem.66.2.783-787.2000. 10653751 PMC91896

[25] Iniguez AL, Dong Y, Triplett EW. 2004. Nitrogen fixation in wheat provided by *Klebsiella pneumoniae* 342. Mol Plant Microbe Interact 17:1078–1085. doi:10.1094/MPMI.2004.17.10.1078. 15497400

[26] Liu S, Hu X, Lohrke SM, Baker CJ, Buyer JS, de Souza JT, Roberts DP. 2007. Role of *sdhA* and *pfkA* and catabolism of reduced carbon during colonization of cucumber roots by *Enterobacter cloacae*. Microbiology (Reading) 153:3196–3209. doi:10.1099/mic.0.2006/005538-0. 17768262

[27] Shankar M, Ponraj P, Ilakkiam D, Gunasekaran P. 2011. Root colonization of a rice growth promoting strain of *Enterobacter cloacae*. J Basic Microbiol 51:523–530. doi:10.1002/jobm.201000342. 21656802

[28] Zurfluh K, Nüesch-Inderbinen M, Morach M, Zihler Berner A, Hächler H, Stephan R. 2015. Extended-spectrum-β-lactamase-producing *Enterobacteriaceae* isolated from vegetables imported from the Dominican Republic, India, Thailand, and Vietnam. Appl Environ Microbiol 81:3115–3120. doi:10.1128/AEM.00258-15. 25724954 PMC4393435

[29] Rossi F. 2011. The challenges of antimicrobial resistance in Brazil. Clin Infect Dis 52:1138–1143. doi:10.1093/cid/cir120. 21467020

[30] Gales AC, Castanheira M, Jones RN, Sader HS. 2012. Antimicrobial resistance among Gram-negative bacilli isolated from Latin America: results from SENTRY Antimicrobial Surveillance Program (Latin America, 2008–2010.). Diagn Microbiol Infect Dis 73:354–360. doi:10.1016/j.diagmicrobio.2012.04.007. 22656912

[31] Sampaio JL, Gales AC. 2016. Antimicrobial resistance in Enterobacteriaceae in Brazil: focus on β-lactams and polymyxins. Braz J Microbiol 47 Suppl 1:31–37. doi:10.1016/j.bjm.2016.10.002. 27825605 PMC5156504

[32] Rocha FR, Pinto VP, Barbosa FC. 2016. The spread of CTX-M-type extended-spectrum β-lactamases in Brazil: a systematic review. Microb Drug Resist 22:301–311. doi:10.1089/mdr.2015.0180. 26669767

[33] Silva KC, Moreno M, Cabrera C, Spira B, Cerdeira L, Lincopan N, Moreno AM. 2016. First characterization of CTX-M-15-producing *Escherichia coli* strains belonging to sequence type (ST) 410, ST224, and ST1284 from commercial swine in South America. Antimicrob Agents Chemother 60:2505–2508. doi:10.1128/AAC.02788-15. 26824955 PMC4808232

[34] Silva MM, Fernandes MR, Sellera FP, Cerdeira L, Medeiros LKG, Garino F, Azevedo SS, Lincopan N. 2018. Multidrug-resistant CTX-M-15-producing *Klebsiella pneumoniae* ST231 associated with infection and persistent colonization of dog. Diagn Microbiol Infect Dis 92:259–261. doi:10.1016/j.diagmicrobio.2018.06.012. 30025966

[35] Nascimento T, Cantamessa R, Melo L, Fernandes MR, Fraga E, Dropa M, Sato MIZ, Cerdeira L, Lincopan N. 2017. International high-risk clones of *Klebsiella pneumoniae* KPC-2/CC258 and *Escherichia coli* CTX-M-15/CC10 in urban lake waters. Sci Total Environ 598:910–915. doi:10.1016/j.scitotenv.2017.03.207. 28458208

[36] Sellera FP, Fernandes MR, Moura Q, Souza TA, Cerdeira L, Lincopan N. 2017. Draft genome sequence of *Enterobacter cloacae* ST520 harbouring *bla*_KPC-2_, *bla*_CTX-M-15_ and *bla*_OXA-17_ isolated from coastal waters of the South Atlantic Ocean. J Glob Antimicrob Resist 10:279–280. doi:10.1016/j.jgar.2017.07.017. 28827199

[37] Sartori L, Fernandes MR, Ienne S, de Souza TA, Gregory L, Cerdeira L, Lincopan N. 2017. Draft genome sequences of two fluoroquinolone-resistant CTX-M-15-producing *Escherichia coli* ST90 (ST23 complex) isolated from a calf and a dairy cow in South America. J Glob Antimicrob Resist 11:145–147. doi:10.1016/j.jgar.2017.10.009. 29111480

[38] Sartori L, Sellera FP, Moura Q, Cardoso B, Cerdeira L, Lincopan N. 2019. Multidrug-resistant CTX-M-15-positive *Klebsiella pneumoniae* ST307 causing urinary tract infection in a dog in Brazil. J Glob Antimicrob Resist 19:96–97. doi:10.1016/j.jgar.2019.09.003. 31520809

[39] Freitas DY, Araújo S, Folador ARC, Ramos RTJ, Azevedo JSN, Tacão M, Silva A, Henriques I, Baraúna RA. 2019. Extended spectrum beta-lactamase-producing gram-negative bacteria recovered from an Amazonian lake near the city of Belém, Brazil. Front Microbiol 10:364. doi:10.3389/fmicb.2019.00364. 30873145 PMC6403167

[40] Goldberg DW, Fernandes MR, Sellera FP, Costa DGC, Loureiro Bracarense AP, Lincopan N. 2019. Genetic background of CTX-M-15-producing *Enterobacter hormaechei* ST114 and *Citrobacter freundii* ST265 co-infecting a free-living green turtle (*Chelonia mydas*). Zoonoses Public Health 66:540–545. doi:10.1111/zph.12572. 30843359

[41] Cerdeira L, Silva KC, Fernandes MR, Ienne S, de Souza TA, de Oliveira Garcia D, Moreno AM, Lincopan N. 2016. Draft genome sequence of a CTX-M-15-producing *Klebsiella pneumoniae* sequence type 340 (clonal complex 258) isolate from a food-producing animal. J Glob Antimicrob Resist 7:67–68. doi:10.1016/j.jgar.2016.07.012. 27664870

[42] Cerdeira L, Monte DFM, Fuga B, Sellera FP, Neves I, Rodrigues L, Landgraf M, Lincopan N. 2020. Genomic insights of *Klebsiella pneumoniae* isolated from a native Amazonian fish reveal wide resistome against heavy metals, disinfectants, and clinically relevant antibiotics. Genomics 112:5143–5146. doi:10.1016/j.ygeno.2020.09.015. 32916256 PMC7758709

[43] Valencia-Bacca J, Silva MM, Cerdeira L, Esposito F, Cardoso B, Muñoz ME, Jiménez-Villegas T, Cardenas-Arias A, Pessoa DAN, Lincopan N. 2020. Detection and whole-genome analysis of a high-risk clone of *Klebsiella pneumoniae* ST340/CG258 producing CTX-M-15 in a companion animal. Microb Drug Resist 26:611–615. doi:10.1089/mdr.2019.0190. 31809242

[44] Casella T, Cerdeira LT, Fernandes MR, Souza TA, Haenni M, Madec JY, Lincopan N, Nogueira MCL. 2017. Draft genome sequence of a CTX-M-15-producing *Escherichia coli* ST345 from commercial chicken meat in Brazil. J Glob Antimicrob Resist 9:124–125. doi:10.1016/j.jgar.2017.04.002. 28559168

[45] Bhilwadikar T, Pounraj S, Manivannan S, Rastogi NK, Negi PS. 2019. Decontamination of microorganisms and pesticides from fresh fruits and vegetables: a comprehensive review from common household processes to modern techniques. Compr Rev Food Sci Food Saf 18:1003–1038. doi:10.1111/1541-4337.12453. 33337007

[46] Olmez H, Temur SD. 2010. Effects of different sanitizing treatments on biofilms and attachment of *Escherichia coli* and *Listeria monocytogenes* on green leaf lettuce. LWT Food Sci Technol 43:964–970. doi:10.1016/j.lwt.2010.02.005.

[47] Coudeyras S, Nakusi L, Charbonnel N, Forestier C. 2008. A tripartite efflux pump involved in gastrointestinal colonization by *Klebsiella pneumoniae* confers a tolerance response to inorganic acid. Infect Immun 76:4633–4641. doi:10.1128/IAI.00356-08. 18644883 PMC2546844

[48] Liu Y, Tang H, Lin Z, Xu P. 2015. Mechanisms of acid tolerance in bacteria and prospects in biotechnology and bioremediation. Biotechnol Adv 33:1484–1492. doi:10.1016/j.biotechadv.2015.06.001. 26057689

[49] Araújo S, Silva IAT, Tacão M, Patinha C, Alves A, Henriques I. 2017. Characterization of antibiotic resistant and pathogenic *Escherichia coli* in irrigation water and vegetables in household farms. Int J Food Microbiol 257:192–200. doi:10.1016/j.ijfoodmicro.2017.06.020. 28668729

[50] Beuchat LR. 2002. Ecological factors influencing survival and growth of human pathogens on raw fruits and vegetables. Microbes Infect 4:413–423. doi:10.1016/S1286-4579(02)01555-1. 11932192

[51] Hartmann A, Locatelli A, Amoureux L, Depret G, Jolivet C, Gueneau E, Neuwirth C. 2012. Occurrence of CTX-M producing *Escherichia coli* in soils, cattle, and farm environment in France. Front Microbiol 3:83. doi:10.3389/fmicb.2012.00083. 22408639 PMC3297819

[52] Cantas L, Shah SQ, Cavaco LM, Manaia CM, Walsh F, Popowska M, Garelick H, Bürgmann H, Sørum H. 2013. A brief multi-disciplinary review on antimicrobial resistance in medicine and its linkage to the global environmental microbiota. Front Microbiol 4:96. doi:10.3389/fmicb.2013.00096. 23675371 PMC3653125

[53] Huang J. 1986. Ultrastructure of bacterial penetration in plants. Annu Rev Phytopathol 24:141–157. doi:10.1146/annurev.py.24.090186.001041.

[54] Hardoim PR, van Overbeek LS, Elsas JD. 2008. Properties of bacterial endophytes and their proposed role in plant growth. Trends Microbiol 16:463–471. doi:10.1016/j.tim.2008.07.008. 18789693

[55] Schaufler K, Nowak K, Düx A, Semmler T, Villa L, Kourouma L, Bangoura K, Wieler LH, Leendertz FH, Guenther S. 2018. Clinically relevant ESBL-producing *K. pneumoniae* ST307 and *E. coli* ST38 in an urban west African rat population. Front Microbiol 9:150. doi:10.3389/fmicb.2018.00150. 29479341 PMC5812336

[56] Schaufler K, Semmler T, Wieler LH, Trott DJ, Pitout J, Peirano G, Bonnedahl J, Dolejska M, Literak I, Fuchs S, Ahmed N, Grobbel M, Torres C, McNally A, Pickard D, Ewers C, Croucher NJ, Corander J, Guenther S. 2019. Genomic and functional analysis of emerging virulent and multidrug-resistant *Escherichia coli* lineage sequence type 648. Antimicrob Agents Chemother 63:e00243-19. doi:10.1128/AAC.00243-19. 30885899 PMC6535536

[57] Mendes RE, Jones RN, Woosley LN, Cattoir V, Castanheira M. 2019. Application of next-generation sequencing for characterization of surveillance and clinical trial isolates: analysis of the distribution of β-lactamase resistance genes and lineage background in the United States. Open Forum Infect Dis 6:S69–S78. doi:10.1093/ofid/ofz004. 30895217 PMC6419912

[58] Peirano G, Pitout JDD. 2019. Extended-spectrum β-lactamase-producing Enterobacteriaceae: update on molecular epidemiology and treatment options. Drugs 79:1529–1541. doi:10.1007/s40265-019-01180-3. 31407238

[59] Dropa M, Lincopan N, Balsalobre LC, Oliveira DE, Moura RA, Fernandes MR, da Silva QM, Matté GR, Sato MI, Matté MH. 2016. Genetic background of novel sequence types of CTX-M-8- and CTX-M-15-producing *Escherichia coli* and *Klebsiella pneumoniae* from public wastewater treatment plants in São Paulo, Brazil. Environ Sci Pollut Res Int 23:4953–4958. doi:10.1007/s11356-016-6079-5. 26782324

[60] Gonçalves LF, de Oliveira Martins-Júnior P, de Melo ABF, da Silva RCRM, de Paulo Martins V, Pitondo-Silva A, de Campos TA. 2016. Multidrug resistance dissemination by extended-spectrum β-lactamase-producing *Escherichia coli* causing community-acquired urinary tract infection in the Central-Western Region, Brazil. J Glob Antimicrob Resist 6:1–4. doi:10.1016/j.jgar.2016.02.003. 27530830

[61] Fernandes MR, Sellera FP, Moura Q, Gaspar VC, Cerdeira L, Lincopan N. 2018. International high-risk clonal lineages of CTX-M-producing *Escherichia coli* F-ST648 in free-roaming cats, South America. Infect Genet Evol 66:48–51. doi:10.1016/j.meegid.2018.09.009. 30227226

[62] Fernandes MR, Sellera FP, Moura Q, Esposito F, Sabino CP, Lincopan N. 2020. Identification and genomic features of halotolerant extended-spectrum-β-lactamase (CTX-M)-producing *Escherichia coli* in urban-impacted coastal waters, Southeast Brazil. Mar Pollut Bull 150:110689. doi:10.1016/j.marpolbul.2019.110689. 31733900

[63] Sellera FP, Fernandes MR, Ruiz R, Falleiros ACM, Rodrigues FP, Cerdeira L, Lincopan N. 2018. Identification of KPC-2-producing *Escherichia coli* in a companion animal: a new challenge for veterinary clinicians. J Antimicrob Chemother 73:2259–2261. doi:10.1093/jac/dky173. 29800301

[64] Gimeno-Garcia E, Andreu V, Boluda R. 1996. Heavy metals incidence in the application of inorganic fertilizers and pesticides to rice farming soils. Environ Pollut 92:19–25. doi:10.1016/0269-7491(95)00090-9. 15091407

[65] Sipter E, Rózsa E, Gruiz K, Tátrai E, Morvai V. 2008. Site-specific risk assessment in contaminated vegetable gardens. Chemosphere 71:1301–1307. doi:10.1016/j.chemosphere.2007.11.039. 18191173

[66] Kananke T, Wansapala J, Gunaratne A. 2014. Heavy metal contamination in green leafy vegetables collected from selected market sites of Piliyandala area, Colombo District, Sri Lanka. AJFST 2:139–144. doi:10.12691/ajfst-2-5-1.

[67] Osaili TM, Al Jamali AF, Makhadmeh IM, Taha M, Jarrar SK. 2016. Heavy metals in vegetables sold in the local market in Jordan. Food Addit Contam Part B Surveill 9:223–229. doi:10.1080/19393210.2016.1181675. 27117608

[68] Araújo WL, Maccheroni W, Jr, Aguilar-Vildoso CI, Barroso PA, Saridakis HO, Azevedo JL. 2001. Variability and interactions between endophytic bacteria and fungi isolated from leaf tissues of citrus rootstocks. Can J Microbiol 47:229–236. doi:10.1139/w00-146. 11315114

[69] Clinical and Laboratory Standards Institute. 2020. Performance standards for antimicrobial susceptibility testing; CLSI supplement M100, 30th ed. Clinical and Laboratory Standards Institute, Wayne, PA, USA.

[70] Clinical and Laboratory Standards Institute. 2018. Performance standards for antimicrobial disk and dilution susceptibility tests for bacteria isolated from animals; CLSI supplement VET08, 4th ed. Clinical and Laboratory Standards Institute, Wayne, PA, USA.

[71] Jarlier V, Nicolas MH, Fournier G, Philippon A. 1988. Extended broad-spectrum beta-lactamases conferring transferable resistance to newer beta-lactam agents in Enterobacteriaceae: hospital prevalence and susceptibility patterns. Rev Infect Dis 10:867–878. doi:10.1093/clinids/10.4.867. 3263690

[72] Bankevich A, Nurk S, Antipov D, Gurevich AA, Dvorkin M, Kulikov AS, Lesin VM, Nikolenko SI, Pham S, Prjibelski AD, Pyshkin AV, Sirotkin AV, Vyahhi N, Tesler G, Alekseyev MA, Pevzner PA. 2012. SPAdes: a new genome assembly algorithm and its applications to single-cell sequencing. J Comput Biol 19:455–477. doi:10.1089/cmb.2012.0021. 22506599 PMC3342519

[73] Coil D, Jospin G, Darling AE. 2015. A5-miseq: an updated pipeline to assemble microbial genomes from Illumina MiSeq data. Bioinformatics 31:587–589. doi:10.1093/bioinformatics/btu661. 25338718

[74] Siguier P, Perochon J, Lestrade L, Mahillon J, Chandler M. 2006. ISfinder: the reference centre for bacterial insertion sequences. Nucleic Acids Res 34:D32–36. doi:10.1093/nar/gkj014. 16381877 PMC1347377

[75] Lam MMC, Wick RR, Wyres KL, Gorrie CL, Judd LM, Jenney AWJ, Brisse S, Holt KE. 2018. Genetic diversity, mobilisation and spread of the yersiniabactin-encoding mobile element ICEKp in Klebsiella pneumoniae populations. Microb Genom 4:e000196. doi:10.1099/mgen.0.000196. PMC6202445 29985125

[76] Wick RR, Heinz E, Holt KE, Wyres KL. 2018. Kaptive Web: user-friendly capsule and lipopolysaccharide serotype prediction for Klebsiella genomes. J Clin Microbiol 56:e00197-18. doi:10.1128/JCM.00197-18. 29618504 PMC5971559

[77] Wyres KL, Wick RR, Gorrie C, Jenney A, Follador R, Thomson NR, Holt KE. 2016. Identification of Klebsiella capsule synthesis loci from whole genome data. Microb Genom 2:e000102. doi:10.1099/mgen.0.000102. 28348840 PMC5359410

[78] Birnboim HC, Doly J. 1979. A rapid alkaline extraction procedure for screening recombinant plasmid DNA. Nucleic Acids Res 7:1513–1523. doi:10.1093/nar/7.6.1513. 388356 PMC342324

[79] Inoue H, Nojima H, Okayama H. 1990. High efficiency transformation of *Escherichia coli* with plasmids. Gene 96:23–28. doi:10.1016/0378-1119(90)90336-P. 2265755

[80] Tanuja R, Bisht SC, Mishra PK. 2013. Ascending migration of endophytic *Bacillus thuringiensis* and assessment of benefits to different legumes of N.W. Himalayas. Eur J Soil Biol 56:56–64. doi:10.1016/j.ejsobi.2013.02.004.

[81] Versalovic J, Koeuth T, Lupski JR. 1991. Distribution of repetitive DNA sequences in eubacteria and application to fingerprinting of bacterial genomes. Nucleic Acids Res 19:6823–6831. doi:10.1093/nar/19.24.6823. 1762913 PMC329316

[82] Miller LG, Kaspar CW. 1994. *Escherichia coli* O157: H7 acid tolerance and survival in apple cider. J Food Prot 57:460–464. doi:10.4315/0362-028X-57.6.460. 31121664

[83] Presser KA, Ratkowsky DA, Ross T. 1997. Modelling the growth rate of *Escherichia coli* as a function of pH and lactic acid concentration. Appl Environ Microbiol 63:2355–2360. doi:10.1128/AEM.63.6.2355-2360.1997. 9172355 PMC168528

[84] Lincopan N, Carmona-Ribeiro AM. 2006. Lipid-covered drug particles: combined action of dioctadecyldimethylammonium bromide and amphotericin B or miconazole. J Antimicrob Chemother 58:66–75. doi:10.1093/jac/dkl153. 16636081

